# Subgenotyping and genetic variability of hepatitis C virus in Palestine

**DOI:** 10.1371/journal.pone.0222799

**Published:** 2019-10-07

**Authors:** Sahar Rayan Da’as, Maysa Azzeh

**Affiliations:** Virology Research Laboratory, Medical Research Center, Al-Quds University, Abu Dies-East Jerusalem, West Bank, Palestine; University of Cincinnati College of Medicine, UNITED STATES

## Abstract

Hepatitis C virus (HCV) is a major cause of liver cirrhosis and hepatocellular carcinoma. Genotyping of HCV is crucial for successful therapy. To determine the HCV subgenotypes circulating in Palestine and to study the genetic variability of their core, we collected 84 serum samples which had tested positive for anti-HCV antibodies. Thirty-seven of these samples came from hemodialysis patients. Serum samples were subjected to viral RNA isolation and amplification of the HCV core gene. Thirty-three of the samples (39%) tested positive for HCV RNA. The HCV subgenotypes circulating in Palestine included 1a, 3a, and 4a, detected in 38%, 25%, and 22% of the samples, respectively. Furthermore, subgenotype 1b was present in three samples (9%), while the rare subgenotype 4v was present in two samples (6%). We identified a number of substitutions in the retrieved HCV core sequences, such as HCV 1b substitutions R70Q and M91L, which some studies have associated with hepatocellular carcinoma risk and poor virological response. In contrast to two previous studies reporting that HCV genotype 4 was predominant in the Gaza strip (present in just over 70% of samples), genotype 4 was detected in only 31% of the samples in our current study, whereas genotype 1 and 3 were present in 69% of samples. These differences may relate to the fact that many of our samples came from the West Bank and East Jerusalem. The co-circulation of different HCV genotypes and subgenotypes in Palestine suggests that subgenotyping prior to treatment is crucial in Palestinian patients.

## Introduction

In 2015, one percent of the world population, or about 71 million people, were estimated to be infected with HCV, with 1.75 million new HCV infections [1]. The predominant modes of HCV transmission were injection drug use and unsafe health-care practices [1]. One of the worst examples of the latter occurred in Egypt in the 1960s to 1980s, when insufficiently sterilized injection equipment use during anti-schistosomiasis treatment resulted in the catastrophic spread of HCV [2–4]. In 2015, the prevalence of antibody to HCV in Egypt was estimated as 10% and that of HCV RNA as 7%, which is the highest in the world [4]. These facts illustrate that despite major advances in prevention, health care standards, diagnostics, and treatment; HCV continues to be a threatening bloodborne pathogen.

Due to the lack of vaccines against HCV, treatment of HCV infection is decisive and is now possible with the new generation of direct-acting antivirals (DAAs). DAAs are HCV-specific, targeting various viral proteins involved in HCV replication. DAAs can result in sustained virologic response (SVR) rates higher than 90%, with minimal adverse effects and high tolerability [3]. Assay of the HCV genotype and subgenotype are recommended before starting DAA antiviral therapy [5, 6]. Indeed, the choice of treatment regimens and duration are most efficient when tailored based on: genotype; subgenotype in case of genotype 1 (1a or 1b); the presence of mixed genotypes; cirrhosis status; and previous treatments [5, 6].

Palestine is part of the region with the highest HCV prevalence worldwide, the Eastern Mediterranean region [1]. While previous studies from Palestine described HCV genotypes circulating in Gaza strip only [7, 8], our study provides the first insight into HCV subgenotypes circulating throughout Palestine (West Bank, East Jerusalem, and Gaza strip) in the general population and in hemodialysis patients, and sheds light on the genetic variability of the core gene of these Palestinian HCV isolates.

## Materials and methods

### Ethics statement and study population

The Al-Quds University ethics committee approved this study (reference number 2/REC/28). The Study sample comprised Palestinian adults from East Jerusalem, the West Bank, and Gaza strip, who had tested positive for anti-HCV antibodies. Testing positive for anti-HCV antibodies was the inclusion criterion for samples. The study samples were either archived at the Virology Research Laboratory (VRL, Al-Quds University), or came from blood donors, patients contacted privately, or hemodialysis patients. The archived samples were residual serum samples from the Al-Makassed Islamic Charitable Hospital (MICH) Central Laboratory and were collected between 2010 and 2013. (These would typically have been discarded, but instead were purposely stored for proposed research at VRL). The use of these archived samples and the collection of further blood donor samples, which occurred in the later years 2015–2016, were approved by the MICH ethics committee. Privately contacted patients were recruited by “word-of-mouth” referral sampling through our friends, relatives, acquaintances, and colleagues, and hemodialysis patients (HD) were recruited through nephrologists (all sources are listed in Acknowledgment). The samples collected between 2015–2016 comprised blood donor samples, privately collected samples, and hemodialysis samples. (According to medical charts, only one of the archived samples belonged to a hemodialysis patient). In summary we collected 84 samples, 37 belonged to hemodialysis patients (HD) and 47 belonged to non-hemodialysis individuals (non-HD).

Every participant signed a consent form before sampling. The consent form contained the following data: sex, age, residency, hemodialysis status, anti-HCV status, and anti-HCV medication intake if any. Additionally, the consent form included questions regarding previous major surgeries, blood transfusions, and kidney transplantation.

### Amplification of the HCV core gene

HCV RNA was extracted from serum samples using the QIAamp Viral RNA Mini kit (Cat. # 52906, Qiagen, Germany). The core gene was amplified in a nested RT-PCR reaction using the One-Step RT-PCR Kit (Cat. # 210212, Qiagen, Germany) and PCR ReddyMix (AB-0575\Dc\LD, ThermoFisher scientific). The specific primers used to amplify the core gene were modified from Lole et al. [9] and indicated in Table 1.

**Table 1:** Primers used to amplify the core gene of HCV isolates.

Primer Name	Primer (location on gene)	Primer sequence (5’- 3’)	PCR Product (bp)
SRcoreF1	288–308	ACT GCC TGA TAG GGY GCT TGC	
SRcoreR1	732–751	ATG TAY CCC ATG AGG TCG GC	464
SRcoreF2	321–339	AGG TCT CGT AGA CCG TGC A	
SRcoreR2	705–724	CAN GTD AGG GTA TCG ATG AC	404

IUPAC nucleotide code: Y = C or T; N = G or A or T or C; D = A or G or T.

### Gene sequence analysis

Sequences were initially subjected to NCBI blast analysis to identify the HCV subgenotypes. (Some type 4, but non-4a subgenotype were additionally subjected to blast analysis using the HCV sequence database for confirmation [10]). Next, the sequences were aligned with NCBI-archived sequences representing the same subgenotype (or different subgenotypes of genotype 4 in case of the non-4a subgenotype, S1 Table) using the MegAlign program (Lasergene, version 15, DNASTARInc., WI, USA). The MegAlign software marked nucleotide differences between the aligned sequences. Subsequently, chromatograms of our sequences were inspected visually for these nucleotides to check if the chromatogram peaks and deduced bases matched. This essential procedure verified that a detected nucleotide substitution was due to real mutation and not to a technical problem in the automatic chromatogram base calling. This procedure also allowed the identification of quasispecies, along with additional inspection of single positions with overlapping peaks in the chromatogram. The annotated Palestinian sequences were submitted to GenBank and assigned the accession numbers MK185615-MK185646. The MegAlign program was also used to generate measures of sequence distances and percent identity among aligned sequences.

### Characterizing identified nucleotide and amino acid substitutions

Some of the nucleotide and amino acid substitution events we identified in the Palestinian isolates were found in previous reports (using PubMed search) or in NCBI-archived sequences (cited in S2–S10 Tables). For the remaining substitutions we found no previous reports (listed as N/A in S2–S10 Tables).

### Phylogenetic tree

The MegAlign program was used to align Palestinian and reference sequences (S1 Table). The phylogenetic tree was generated within the program Mega 7 (https://www.megasoftware.net) using the Neighbor-Joining method and with 1,000 bootstrap replicates; only bootstrap values over 50 were shown.

## Results

### Demographics of study sample

The study samples were from individuals residing in Gaza Strip (n = 10), the West Bank (n = 52), and East Jerusalem (n = 22). The male to female ratio was 1.27, while the overall median age was 50.5 (range 20–80 years old).

### Samples with positive HCV RNA by region and medical records

A total of 33 (39.3%) out of the 84 samples tested positive for HCV RNA, five from Gaza, 18 from the West bank, and 10 from East Jerusalem, respectively. Of the HCV RNA positive sample, 12 came from the hemodialysis patient group (HD), and 21 came from the non-HD patients. (The HCV RNA negative samples might partially reflect false positives from the HCV antibody test, because we did not have the resources to retest the samples for HCV antibodies).

Most of the HD patients (25/37, or 67.5%) stated they seroconverted after starting dialysis. Furthermore, the majority of HD patients had received at least one blood transfusion (26/37, or 70%). Blood transfusions occurred mainly at local Palestinian hospitals.

Only 10 patients of the total study sample received antiviral treatment, defined as at least one course of PEG-interferon and Ribavirin (PEG-IFN/RBV) antiviral therapy, before our sampling.

The proportions of samples testing positive for HCV RNA were not skewed towards any particular diagnosis or treatment history. For examples, five of the 10 patients who had received the antiviral treatment tested positive for HCV RNA. Though HCV RNA was detected in 38.5% of patients that had received blood transfusions (10/26) and in only 18% (2/11) of those that had received no blood transfusion, this difference was not significant (p-value = 0.3).

Eight HD patients had a history of kidney transplantation (samples from four of these patients tested positive for HCV RNA). Two of these received the kidney transplantations in Pakistan (with kidneys from Pakistanis); the third had the kidney transplantation in Iraq (with a kidney from an Egyptian). In the fourth case, the patient received two kidney transplantations within two years, the first in India (with a kidney from an Indian) and the second in Egypt (with a kidney from an Egyptian).

### Identification of HCV subgenotypes circulating in Palestine

**Fig 1:**
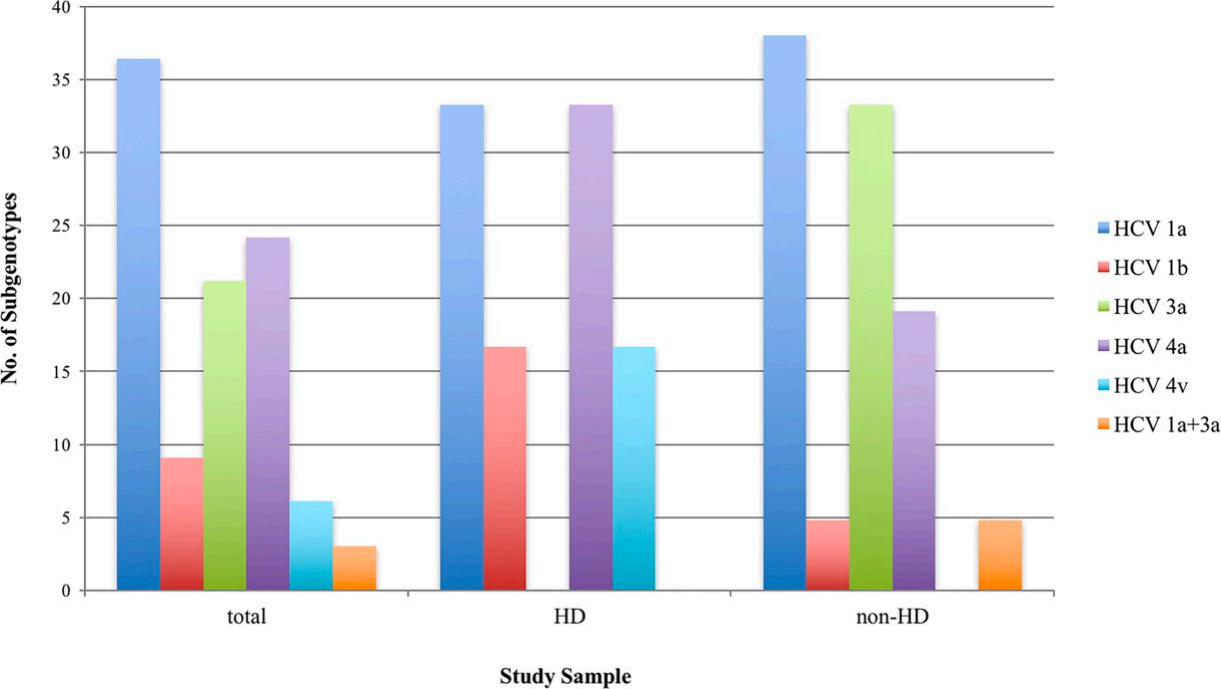
Distribution of HCV subgenotypes in the Palestinian study sample. Total represents the overall distribution of subgenotypes in the 33 samples, HD represents the distribution of subgenotypes in the hemodialysis patients, and non-HD represents the subgenotypes in the non-hemodialysis group.

The sequence analysis of the core genes amplified from 33 HCV RNA-positive Palestinian serum samples revealed five different HCV subgenotypes, belonging to genotypes 1, 3, and 4. HCV genotype 1 was detected in 47% of the samples and represented by subgenotypes 1a (38%) and 1b (9%). HCV genotype 4 was detected in 31% and represented by subgenotypes 4a (25%) and 4v (6%). Finally, HCV genotype 3 was detected in 22% of the samples and represented only by subgenotype 3a (Fig 1). One sample contained a mix of HCV subgenotypes 1a and 3a.

The distribution of genotypes and subgenotypes in the HD and non-HD group is shown in Fig 1. Genotypes 1, 3, and 4 were detected in the non-HD group, while only genotypes 1 and 4 were found in the HD patients. Subgenotype 1b was detected in both the HD and the non-HD group, while the rare subgenotype 4v was only detected in the HD patient group. These distribution differences were not statistically significant.

The above-mentioned sample with a mix of subgenotypes 1a and 3a was from a non-HD patient (Fig 1). One 4v isolate came from an archived sample, which belonged to a HD patient and was collected in 2010 (see materials and methods section). The second 4v isolate was collected in a HD patient in 2016.

The four kidney transplantation HD patients who were HCV RNA-positive presented with subgenotypes 1a, 4v, and 4a. Specifically, 4a was detected in the patient who received a kidney from an Egyptian in Iraq (accession number MK185642), and in the patient who was transplanted twice, in India and in Egypt (MK185644). 1a and 4v were detected in one each of the two patients who received transplantations in Pakistan (1a, MK185623 and 4a, MK185646). All four kidney transplant HD patients received at least one blood transfusion in Palestine ~10–30 years after transplantations, specifically when the transplanted kidney failed and they were back on hemodialysis.

**Fig 2:**
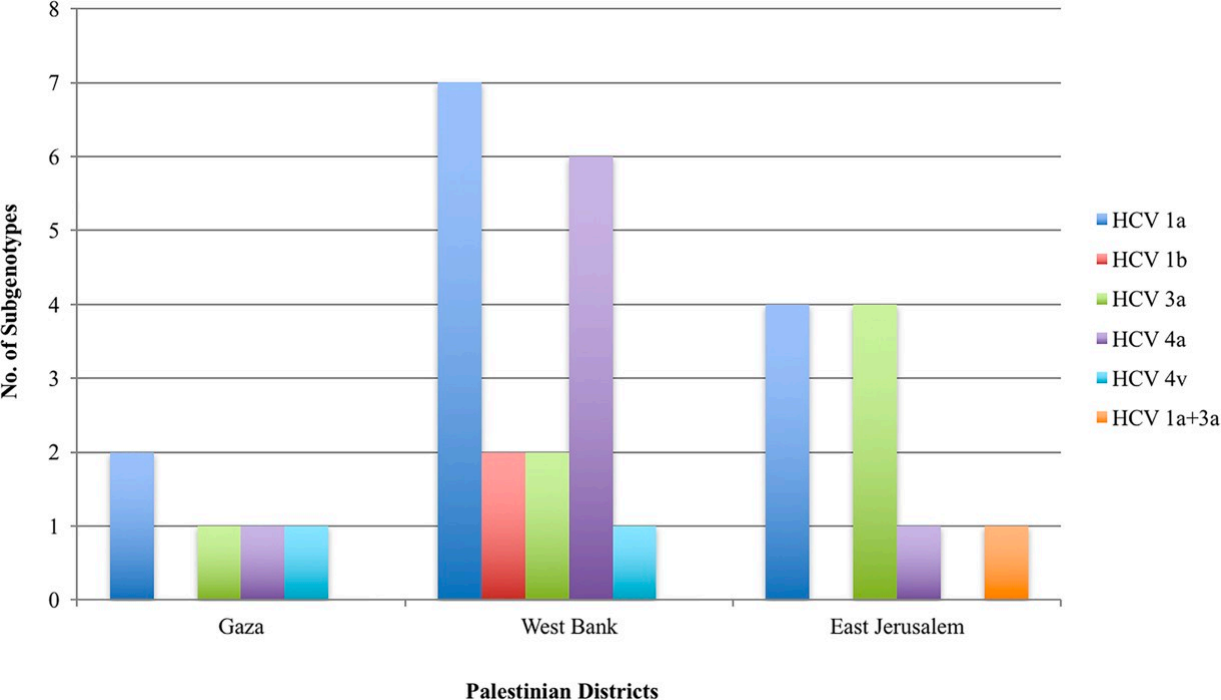
Distribution of HCV subgenotypes of study sample in Palestinian districts. The number of the different subgenotypes detected in each district are indicated.

In regards to distribution of genotypes within specific regions in Palestine, genotypes 3 and 1 distributed equally in East Jerusalem, genotype 1 and 4 distributed almost equally in the West Bank, and genotypes 1 and 4 distributed equally in Gaza, where genotype 3 was also detected (Fig 2). However, these regional differences were not statistically significant.

In the five HCV RNA-positive samples from patients who received PEG-IFN/RBV antiviral therapy, the subgenotypes detected were 1a (n = 1), 1b (n = 1), and 4a (n = 3).

### Occurrence of quasispecies in Palestinian HCV isolates

Many of the HCV isolates included the subtly different subpopulations of RNA sequences that indicate the presence of viral quasispecies. Our data suggest that quasispecies were present in six of the 1a samples, the three 1b samples, three of the 3a samples, and two of the 4a samples (substitution variants due to quasispecies are marked with asterisks in S2–S9 Tables). These data suggest that quasispecies were present in many treatment naïve patients, because only five of the 33 HCV RNA-positive samples came from patients that had undergone treatment (see Results above).

### Substitutions in Palestinian HCV isolates

Eight non-synonymous and 27 synonymous substitutions were detected in the core genes of the Palestinian HCV 1a isolates (S2 and S3 Tables). Three of the non-synonymous substiutions A145G/A (T49A), A223A/G (T75A), and G272A (C91Y) were previously reported [11–14].

Five non-synonymous and 17 synonymous substitutions were detected in the Palestinian HCV 1b isolates (S4 and S5 Tables). Three of the non-synonymous substitutions A28C (K10Q), G209A/G (R70Q), and A271T (M91L) were previously reported [15–18].

Ten non-synonymous and 11 synonymous substitutions were recorded in the HCV 3a Palestinian isolates (S6 and S7 Tables). A59C (Q20P), G209A, and G210A (R70Q) were detected in previous reports [11, 19].

We recorded five non-synonymous and 19 synonymous substitutions in the Palestinian HCV 4a isolates (S8 and S9 Tables). Substitutions C8T (T3M) and G179A (G60E) were detected in previous research studies [20].

In the two HCV 4v Palestinian isolates we detected no non-synonymous substitutions and five synonymous substitutions (S10 Table).

### Phylogenetic analysis of the Palestinian HCV isolates

**Fig 3:**
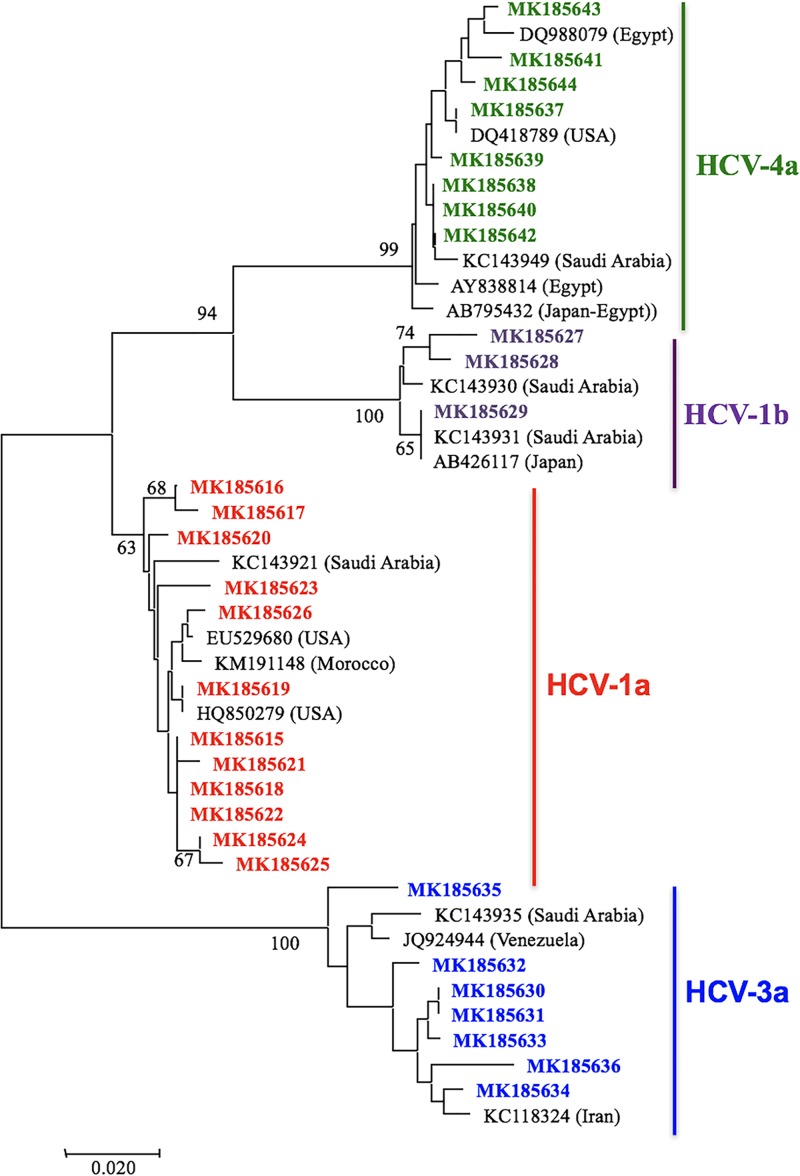
Neighbor-Joining phylogenetic analysis of Palestinian HCV 1a, 1b, 4a, and 3a isolates. Palestinian 1a (red), 1b (purple), 3a (blue), and 4a (green) HCV isolates were compared with regional and international isolates. Origin of NCBI-archived subgenotypes is indicated in parentheses.

A neighbor-joining phylogenetic tree (Fig 3) was created using 1a, 1b, 3a, and 4a isolates from Palestinian sequences and some NCBI-archived sequences (S1 Table).

The percent identity among Palestinian 4a isolates ranged from 96.3% to 100%. Three of the Palestinian 4a isolates from the West Bank and Jerusalem, MK185638, MK185640, and MK185642 (from one of the kidney transplant patients) were almost identical and clustered with KC143949 from Saudi Arabia. MK185643 clustered with DQ988079 from Egypt and MK185637 clustered with DQ418789 from the USA. The 4a isolate from the other kidney transplant patient, MK185644, showed 97.3%-97.9% percent identity with the other Palestinian 4a isolates.

The percent identity among Palestinian 1b isolates ranged from 96% to 99.2%. Palestinian 1b isolates MK185627 and MK185628 sub-clustered from a branch holding KC143930 from Saudi Arabia. MK185629 clustered on one branch with KC143931 from Saudi Arabia and AB426117 from Japan.

The percent identity among Palestinian 1a isolates ranged from 97.6% to 100%, with five almost identical samples from East Jerusalem, Gaza, and the West Bank (MK185615, MK185616, MK185618, MK185620, and MK185622). MK185619 clustered with HQ850279 from the USA. The 1a isolate of the kidney transplant, MK185623, showed 98.4% to 99.2% percent identity with the other Palestinian 1a isolates.

Percent identity among Palestinian 3a isolates ranged from 95% to 100%. Palestinian 3a isolates MK185630 from Gaza and MK185631 from East Jerusalem were identical. MK185634A clustered on one branch with KC1183243 from Iran.

**Fig 4:**
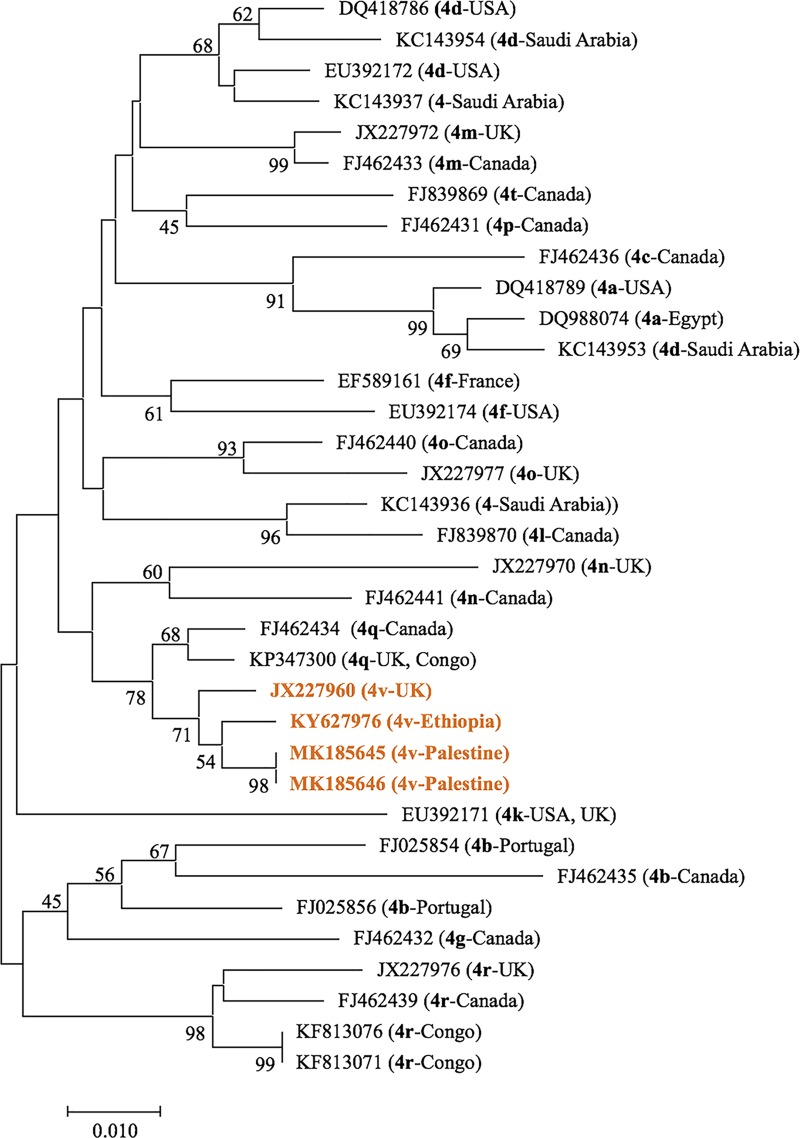
Neighbor-Joining phylogenetic analysis of Palestinian HCV 4v isolates. Palestinian 4v isolates were compared with NCBI-archived 4a, 4b, 4c, 4d, 4f, 4g, 4k, 4l, 4m, 4n, 4o, 4p, 4q, 4r, 4t, 4v, and unknown type 4 subgenotypes. The Palestinian 4v isolates and the other two NCBI-archived 4v isolates are colored orange. Origin of NCBI-archived subgenotypes is indicated in parentheses.

A neighbor-joining phylogenetic tree with various type 4 subgenotypes was generated to verify the two Palestinian 4v isolates (Fig 4). Our two 4v isolates (MK185645 from Gaza and MK185646 from a kidney transplant in the West Bank) were identical and clustered with 4v isolates KY627976 from Ethiopia and JX227960 from the UK.

## Discussion

This is the first study tackling molecular epidemiology and genetic diversity of HCV circulating among Palestinians in the West Bank, Gaza Strip, and East Jerusalem.

In regard to genotype, our results revealed that HCV genotypes 1, 3, and 4 were co-circulating in Palestine, but did not establish predominance of one type. Previous reviews concluded that genotype 4 was the most prevalent in Palestine [21, 22]. However, these reviews were based on the two studies involving only Gaza Strip Palestinians, in which genotype 4 was present in approximately 70% of samples (18/23 and 66/92 samples, respectively) [7, 8]. Our findings may reflect a mix of regional influences, because previous studies showed HCV genotype 4 was dominant in countries such as Jordan, Egypt, and Saudi Arabia, and HCV genotype 1 was the most prevalent in Algeria, Israel, Turkey, and Iran (reviewed in [21, 22]). Genotype 3 was also reported in most countries in the region [21].

The subgenotypes we found also suggest a mix of regional influences, though subgenotypes have been less commonly reported in the region. For example, for genotype 1, subgenotype 1a was in twelve of our samples and 1b was in only three samples, similar to reports that 1a was the predominant subtype of genotype 1 found in Lebanon and UAE [23, 24]. In contrast, 1b was dominant in Israel, Turkey, and the Maghreb region [25–27]. Subgenotype 4a was the predominant subtype of genotype 4 in Egypt [28, 29] and was detected in eight of our samples. Based on our data analysis, one of these eight Palestinian HCV 4a infections was acquired in the Middle East outside Palestine via kidney transplantation. Subtype 3a was reported in UAE and Bahrain [24, 30] and was detected in seven of our samples. Our 4v isolates are the first reported in the region; according to the GenBank there are another nine 4v isolates in the world, which came mainly from Africa.

The subgenotypes isolated from the four kidney transplant patients (all of whom were also blood transfusion recipients in Palestine) were 1a, 4a (two patients), and 4v, consistent with the variety of subgenotypes we found in the other Palestinian samples. Based on the percent identity between two of these isolates (4v, MK185646 and 4a, MK185642) and the other Palestinian isolates of the same subgenotype, which was 99.7%-100%, we propose that the infection occurred in Palestine. The evidence for a Palestinian source of infection is less conclusive for the two other transplant patients with subgenotypes 1a (MK185623) and 4a (MK185644) because these isolates had lower percent identity with our other Palestinian samples suggesting that the infection occurred outside Palestine (see results and Fig 3).

Some of the substitutions we detected in Palestinian HCV isolates have been previously associated with HCC risk in some reports (reviewed in S2–S10 Tables). Two of these substitutions, R70Q and M91L, were supported by several reports. R70Q and M91L were detected in one Palestinian 1b isolate and belonged to one of the few patients who had received Peg-IFN-plus-RBV therapy. R70Q and M91L in 1b isolates were associated with increased HCC risk and poor virological response [15–18, 31–33], although the link between these substitutions and interferon responsiveness or HCC is not firmly established. Furthermore, R70Q was considered a pretreatment predictor of posttreatment HCC, because the presence of R70Q at the start of antiviral therapy was associated with later HCC diagnosis, years after achieving SVR and eradication of HCV RNA, by use of Peg-IFN-plus-RBV or DAA therapy [34–37].

Our study demonstrated, that different HCV subgenotypes, 1a, 1b, 3a, 4a, and 4v are co-circulating in Palestine within different population groups including hemodialysis patients. Additionally, we shed light on the substitutions detected in these isolates, including those previously associated with HCC risk. Based on these findings we advocate for subgenotyping to be prerequisite to therapy in Palestine to achieve sustained virologic response in Palestinian HCV patients.

## Supporting information

S1 Table NCBI-archived reference sequences of complete HCV genomes or HCV core genes used for the substitution analysis and phylogenetic tree. (DOCX)

S2 Table Non-synonymous substitutions in the HCV core gene detected in Palestinian HCV isolates of subgenotype 1a (n = 12). (DOCX)

S3 Table Synonymous substitutions in the HCV core gene detected in Palestinian HCV isolates of subgenotype 1a (n = 12). (DOCX)

S4 Table Non-synonymous substitutions detected in the HCV core gene in Palestinian HCV isolates of subgenotype 1b (n = 3). (DOCX)

S5 Table Synonymous substitutions detected in the HCV core gene in Palestinian HCV isolates of subgenotype 1b (n = 3). (DOCX)

S6 Table Non-synonymous substitutions detected in the HCV core gene in Palestinian HCV isolates of subgenotype 3a (n = 7). (DOCX)

S7 Table Synonymous substitutions detected in the HCV core gene in Palestinian HCV isolates of subgenotype 3a (n = 7). (DOCX)

S8 Table Non-synonymous substitutions detected in the HCV core gene in Palestinian HCV isolates of subgenotype 4a (n = 8). (DOCX)

S9 Table Synonymous substitutions detected in the HCV core gene in Palestinian HCV isolates of subgenotype 4a (n = 8). (DOCX)

S10 Table Synonymous substitutions detected in the HCV core gene in Palestinian HCV isolates of subgenotype 4v (n = 2). (DOCX)
