# Supplementary material for: Subgenotyping and genetic variability of hepatitis C virus in Palestine
Source: PLoS One. 2019 Oct 7;14(10):e0222799. doi: 10.1371/journal.pone.0222799 (PMC6779298; doi:10.1371/journal.pone.0222799)
Supplement: S1 Table — (DOCX) [file pone.0222799.s001.docx]

**S1 Table. NCBI-archived reference sequences of complete HCV genomes or HCV core genes used for the substitution analysis and phylogenetic tree.**

| **Subgenotype** | **Accession No.** | **Country of Isolation** | **Collection date** |
| --- | --- | --- | --- |
| 1a | EU529680 | USA | 2008 |
| 1a | EU255952 | Switzerland | 2007 |
| 1a | EU781801 | Boston | 2006 |
| 1a | EU781800 | Boston | 2006 |
| 1a | AF290978 | USA | 2000 |
| 1a | FJ390399 | USA | 2007 |
| 1a | EF407450 | USA | 2007 |
| 1a | KC143921 | Saudi Arabia | 2009 |
| 1a | KM191148 | Morocco | 2014 |
| 1a | KM261796 | Morocco | 2014 |
| 1a | KC143869 | Saudi Arabia | 2009 |
| 1a | EF407457 | USA | 2007 |
| 1a | HQ850279 | USA | 2011 |
| 1a | KT735187 | France | 2014 |
| 1a | NC_004102 | USA | 1997 |
| 1b | KC143931 | Saudi Arabia | 2009 |
| 1b | KC143930 | Saudi Arabia | 2009 |
| 1b | KC143886 | Saudi Arabia | 2009 |
| 1b | KC143880 | Saudi Arabia | 2009 |
| 1b | AB426117 | Japan | 2008 |
| 1b | KT735184 | France | 2010 |
| 1b | GU441385 | Indonesia | 2007 |
| 1b | KT983617 | Russia | 2015 |
| 1b | KC118292 | Iran | 2013 |
| 1b | KC118309 | Iran | 2013 |
| 1b | EU781827 | USA | 2008 |
| 1b | EU781828 | USA | 2008 |
| 3a | KC143935 | Saudi Arabia | 2009 |
| 3a | KC143897 | Saudi Arabia | 2009 |
| 3a | KC118324 | Iran | 2013 |
| 3a | KC118329 | Iran | 2013 |
| 3a | EU435145 | Pakistan | 2008 |
| 3a | KC796013 | Pakistan | 2012 |
| 3a | JQ924946 | Venezuela | 2006 |
| 3a | JQ924944 | Venezuela | 2001 |
| 3a | JQ717260 | India | 2008 |
| 3a | KC844041 | China | 2011 |
| 3a | KF035125 | India | 2012 |
| 3a | X76918 | Germany | 1993 |
| 3a | JN714194 | India | 2001 |
| 4a | KC143952 | Saudi Arabia | 2009 |
| 4a | KC143949 | Saudi Arabia | 2009 |
| 4a | DQ988079 | Egypt | 2006 |
| 4a | DQ988078 | Egypt | 2006 |
| 4a | AB795432 | Japan (Egypt) | 2012 |
| 4a | GU814265 | Denmark (Egypt) | 2010 |
| 4a | GU814266 | Egypt | 2010 |
| 4a | AY838815 | Egypt | 2004 |
| 4a | AY838814 | Egypt | 2004 |
| 4a | NC_009825 | UK | 1997 |
| 4a | KC118333 | Iran | 2012 |
| 4a | DQ988074 | Egypt | 2006 |
| 4a | DQ418789 | Boston | 2006 |
| 4v | JX227960 | United Kingdom | - |
| 4v | JX227959 | United Kingdom | - |
| 4v | KY627976 | Ethiopia | 2013-2014 |
| 4q | FJ462434 | Canada | 2002-2007 |
| 4q | KP347300 | UK (Congo) | 2012 |
| 4n | JX227970 | United Kingdom | - |
| 4n | FJ462441 | Canada | 2002-2007 |
| 4m | JX227972 | United Kingdom | - |
| 4m | FJ462433 | Canada | 2002-2007 |
| 4d | DQ418786 | USA | 2006 |
| 4d | EU392172 | USA | 2008 |
| 4c | FJ462436 | Canada | 2002-2007 |
| 4a | DQ418789 | USA | 2006 |
| 4a | DQ988074 | Egypt | 2006 |
| 4o | FJ462440 | Canada | 2002-2007 |
| 4o | JX227977 | United Kingdom | - |
| 4t | FJ839869 | Canada | 2002-2007 |
| 4p | FJ462431 | Canada | 2002-2007 |
| 4f | EF589161 | France | 2007 |
| 4f | EU392174 | USA | 2008 |
| 4b | FJ025856 | Portugal | 2008 |
| 4b | FJ025854 | Portugal | 2008 |
| 4r | JX227976 | United Kingdom | 2013 |
| 4k | EU392171 | USA (UK) | 2008 |
| 4r | KF813076 | Congo | 2007 |
| 4d | KC143954 | Saudi Arabia | 2014 |
| 4d | KC143953 | Saudi Arabia | 2014 |
| 4 | KC143937 | Saudi Arabia | 2014 |
| 4 | KC143936 | Saudi Arabia | 2014 |
| 4r | KF813071 | Congo | 2007 |
| 4r | FJ462439 | Canada | 2002-2007 |
| 4l | FJ839870 | Canada | 2002-2007 |
| 4b | FJ462435 | Canada | 2002-2007 |
| 4g | FJ462432 | Canada | 2002-2007 |
