# Supplementary material for: Subgenotyping and genetic variability of hepatitis C virus in Palestine
Source: PLoS One. 2019 Oct 7;14(10):e0222799. doi: 10.1371/journal.pone.0222799 (PMC6779298; doi:10.1371/journal.pone.0222799)
Supplement: S2 Table — (DOCX) [file pone.0222799.s002.docx]

**S2 Table. Non-synonymous Substitutions in the HCV core gene detected in Palestinian HCV isolates of subgenotype 1a (n=12).**

| **Substitution**  **nt** | **Substitution**  **aa** | **N** | **Reference** | **Function in reference** |
| --- | --- | --- | --- | --- |
| C44T | T15I | 1 | N/A |  |
| A145G/A* | T49A | 2 | [11, 12] | Associated with reduced core Antigen levels |
| A152G | K51R | 1 | N/A |  |
| T194C | I65T | 2 | N/A |  |
| A223A/G* | T75A | 1 | [13] | Associated with absence of anti-core antibodies |
| A223A/G/T* | T75A  T75S | 1 | A223T: EF407450 |  |
| G229A | A77T | 1 | N/A |  |
| G272A | C91Y | 1 | [14] | Associated with HCC risk |

*: Substitution base variants, consistent with quasispecies population. N: Number of Palestinian isolates exhibiting the substitution.
