# Supplementary material for: Subgenotyping and genetic variability of hepatitis C virus in Palestine
Source: PLoS One. 2019 Oct 7;14(10):e0222799. doi: 10.1371/journal.pone.0222799 (PMC6779298; doi:10.1371/journal.pone.0222799)
Supplement: S3 Table — (DOCX) [file pone.0222799.s003.docx]

**S3 Table. Synonymous Substitutions in the HCV core gene detected in Palestinian HCV isolates of subgenotype 1a (n=12).**

| **Substitution**  **nt** | **Substitution**  **aa** | **N** | **Reference** | **Function in reference** |
| --- | --- | --- | --- | --- |
| C66T  C66C/T* | V22V  V22V | 3  1 | 13 | N/A |
| T78C | G26G | 1 | 11 | Associated with HCC risk |
| T84A  T84A/T* | G28G  G28G | 2  2 | KC143921 | N/A |
| T126C  T126C/T* | P42P  P42P | 2  1 | 11 | Potentially associated with HCC risk |
| A129G  A129A/G* | R43R  R43R | 1  2 | KM261796 | N/A |
| A183A/G* | R61R | 1 | 11 | Potentially associated with HCC risk |
| T186T/C* | R62R | 1 | N/A | N/A |
| T192C/T* | P64P | 1 | N/A | N/A |
| A204G  A204G/A* | A68A  A68A | 8  1 | 13 | N/A |
| C213T/C* | P71P | 1 | N/A | N/A |
| T231A/G* | A77A | 1 | T231A: FJ390399 | N/A |
| T231T/C/A* | A77A | 1 | T231C: EU781801 | N/A |
| T258C  T258C/T* | Y86Y  Y86Y | 1  1 | EU529680 | N/A |
| T264C  T264T/C* | N88N  N88N | 2  1 | 13 | N/A |
| C270T | G90G | 1 | 13 | N/A |
| C273T | C91C | 1 | EU529680 | N/A |
| G282A/G* | A94A | 2 | 11 | Potentially associated with HCC risk |
| T297C  T297C/T* | S99S  S99S | 8  2 | 13 | Associated with absence of anti-core antibodies |
| T303C  T303C/T* | R101R  R101R | 2  1 | 13 | Associated with absence of anti-core antibodies |
| T309C/T* | S103S | 1 | 13 | Associated with absence of anti-core antibodies |
| C318T | S106S | 5 | 13 | Associated with absence of anti-core antibodies |
| C327T | P109P | 1 | 13 | N/A |
| A330T | T110T | 1 | N/A | N/A |
| T342C  T342C/T* | R114R  R114R | 1  1 | EU255952 | N/A |
| G348A | S116S | 1 | N/A | N/A |
| T354T/C* | N118N | 1 | EU529680 | N/A |
| T378T/C* | L126L | 1 | N/A | N/A |

*: Substitution base variants, consistent with quasispecies population. N: number of Palestinian isolates exhibiting the substitution.
