# Supplementary material for: Subgenotyping and genetic variability of hepatitis C virus in Palestine
Source: PLoS One. 2019 Oct 7;14(10):e0222799. doi: 10.1371/journal.pone.0222799 (PMC6779298; doi:10.1371/journal.pone.0222799)
Supplement: S4 Table — (DOCX) [file pone.0222799.s004.docx]

**S4 Table. Non-synonymous Substitutions detected in the HCV core gene in Palestinian HCV isolates of subgenotype 1b (n=3).**

| **Substitution**  **nt** | **Substitution**  **aa** | **N** | **Reference** | **Function in reference** |
| --- | --- | --- | --- | --- |
| A28C | K10Q | 2 | [15] | Increased HCC risk |
| G61A | D21N | 1 | N/A | N/A |
| G209A/G* | R70Q | 1 | [15-18] | Increased HCC risk |
| G223A | A75T | 3 | KT983617 | N/A |
| A271T | M91L | 1 | [15-18] | Increased HCC risk |

*: Substitution base variants, consistent with quasispecies population. N: Number of Palestinian isolates exhibiting the substitution.
