# Supplementary material for: Subgenotyping and genetic variability of hepatitis C virus in Palestine
Source: PLoS One. 2019 Oct 7;14(10):e0222799. doi: 10.1371/journal.pone.0222799 (PMC6779298; doi:10.1371/journal.pone.0222799)
Supplement: S5 Table — (DOCX) [file pone.0222799.s005.docx]

**S5 Table. Synonymous Substitutions detected in the HCV core gene in Palestinian HCV isolates of subgenotype 1b (n=3).**

| **Substitution**  **nt** | **Substitution**  **aa** | **N** | **Reference** | **Function in reference** |
| --- | --- | --- | --- | --- |
| C42C/T* | N14N | 1 | N/A | N/A |
| C66C/T* | V22V | 1 | C66T: 18 | Increased risk of HCC |
| T81T/C* | G27G | 1 | T81C: KT983617 | N/A |
| T93C | V31V | 1 | EU781827 | N/A |
| G162A | E54E | 1 | N/A | N/A |
| G210A | R70R | 2 | KC143931 | N/A |
| C237C/T* | P79P | 1 | N/A | N/A |
| T246C | P82P | 1 | KC143930 | N/A |
| T258C | Y86Y | 1 | KT983617 | N/A |
| C261A/G* | G87G | 1 | N/A | N/A |
| T264C | N88N | 1 | 12 | Associated with HCC risk |
| G276G/A* | G92G | 1 | N/A | N/A |
| T303C  T303C/T* | R101R  R101R | 1  1 | 12 | Associated with decreased HCC risk |
| T309C | S103S | 1 | 12, 13 | Increased risk of HCC |
| C324T/C* | G108G | 1 | N/A | N/A |
| G330T/G* | T110T | 1 | G330T: KC118309 | N/A |
| T354C | N118N | 2 | N/A | N/A |

*: Substitution base variants, consistent with quasispecies population. N: number of Palestinian isolates exhibiting the substitution.
