# Supplementary material for: Subgenotyping and genetic variability of hepatitis C virus in Palestine
Source: PLoS One. 2019 Oct 7;14(10):e0222799. doi: 10.1371/journal.pone.0222799 (PMC6779298; doi:10.1371/journal.pone.0222799)
Supplement: S6 Table — (DOCX) [file pone.0222799.s006.docx]

**S6 Table. Non-synonymous Substitutions detected in the HCV core gene in Palestinian HCV isolates of subgenotype 3a (n=7).**

| **Substitution**  **nt** | **Substitution**  **aa** | **N** | **Reference** | **Function in reference** |
| --- | --- | --- | --- | --- |
| A59C | Q20P | 1 | [11] | N/A |
| G106T | V36L | 4 | N/A | N/A |
| G179A | G60E | 1 | [11] | N/A |
| C181T | R61W | 2 | N/A | N/A |
| G209A  G209A/G* | R70Q | 1  1 | [11, 19] | N/A |
| G210A | R70Q | 1 | [11, 19] | N/A |
| C301A  C301A/C* | R101S  R101S/R | 4  1 | N/A | N/A |
| A328T | N110S | 1 | KC118329 | N/A |
| A329C | N110S | 1 | KC118329 | N/A |
| A329C  A329C/A* | N110T  N110T/N | 2  1 | N/A | N/A |

*: Substitution base variants, consistent with quasispecies population. N: Number of Palestinian isolates exhibiting the substitution.
