# Supplementary material for: Subgenotyping and genetic variability of hepatitis C virus in Palestine
Source: PLoS One. 2019 Oct 7;14(10):e0222799. doi: 10.1371/journal.pone.0222799 (PMC6779298; doi:10.1371/journal.pone.0222799)
Supplement: S7 Table — (DOCX) [file pone.0222799.s007.docx]

**S7 Table. Synonymous Substitutions detected in the HCV core gene in Palestinian HCV isolates of subgenotype 3a (n=7).**

| **Substitution**  **nt** | **Substitution**  **aa** | **N** | **Reference** |
| --- | --- | --- | --- |
| C66T  C66C/T* | V22V | 6  1 | KC143935 |
| A129G | R43R | 1 | KC143935 |
| G144A  G144A/G* | A48A | 5  1 | KC118329 |
| T159C | S53S | 2 | JQ717260 |
| T159A | S53S | 1 | KC143897 |
| A162G | E54E | 1 | KC143897 |
| A186T | R62R | 1 | JQ924944 |
| G210C | R70R | 5 | N/A |
| T231C | A77A | 1 | KC143935 |
| C303T | R101R | 1 | KF035125 |
| T355C/T* | L119L | 1 | EU435145 |

*: Substitution base variants, consistent with quasispecies population. N: number of Palestinian isolates exhibiting the substitution.
