# Supplementary material for: Subgenotyping and genetic variability of hepatitis C virus in Palestine
Source: PLoS One. 2019 Oct 7;14(10):e0222799. doi: 10.1371/journal.pone.0222799 (PMC6779298; doi:10.1371/journal.pone.0222799)
Supplement: S8 Table — (DOCX) [file pone.0222799.s008.docx]

**S8 Table. Non-synonymous Substitutions detected in the HCV core gene in Palestinian HCV isolates of subgenotype 4a (n=8).**

| **Substitution**  **nt** | **Substitution**  **aa** | **N** | **Reference** | **Function in reference** |
| --- | --- | --- | --- | --- |
| C8T | T3M | 2 | [20] | Detected in patients with PEG-IFN/RBV SVR |
| G64A | V22I | 2 | N/A | N/A |
| G149G/A* | R50R/Q | 1 | N/A | N/A |
| G179A | G60E | 2 | [20] | Detected in patients with PEG-IFN/RBV SVR |
| C184C/T* | R62R/C | 1 | N/A | N/A |

*: Substitution base variants, consistent with quasispecies population. N: Number of Palestinian isolates exhibiting the substitution.
