# Supplementary material for: Subgenotyping and genetic variability of hepatitis C virus in Palestine
Source: PLoS One. 2019 Oct 7;14(10):e0222799. doi: 10.1371/journal.pone.0222799 (PMC6779298; doi:10.1371/journal.pone.0222799)
Supplement: S9 Table — (DOCX) [file pone.0222799.s009.docx]

**S9 Table. Synonymous Substitutions detected in the HCV core gene in Palestinian HCV isolates of subgenotype 4a (n=8).**

| **Substitution** | **Substitution**  **aa** | **N** | **Reference** |
| --- | --- | --- | --- |
| T66C  T66C/T* | V22V | 4  1 | KC143952 |
| C84T | G28G | 4 | KC143952 |
| C96C/T* | G32G | 1 | N/A |
| C127A | R43R | 3 | DQ418789 |
| G149G/A* | R50R/Q | 1 | N/A |
| T156T/C* | T52T | 1 | N/A |
| A231T | A77A | 1 | N/A |
| G234A | Q78Q | 3 | GU814265 |
| T243C | Y81Y | 2 | DQ988074 |
| T252C | P84P | 2 | KC143952 |
| T261C | G87G | 2 | KC143952 |
| T270C | G90G | 3 | DQ988078 |
| A285G | G95G | 1 | KC143952 |
| T303C | R101R | 3 | KC143949 |
| C306T | G102G | 5 | DQ988078 |
| A312G | R104R | 2 | KC143949 |
| A327T  A327G/A/T* | P109P  P109P | 1  1 | N/A |
| T355C | L119L | 3 | KC143952 |
| G363A | K121K | 1 | KC118333 |

*: Substitution base variants, consistent with quasispecies population. N: number of Palestinian isolates exhibiting the substitution.
