# Supplementary material for: Subgenotyping and genetic variability of hepatitis C virus in Palestine
Source: PLoS One. 2019 Oct 7;14(10):e0222799. doi: 10.1371/journal.pone.0222799 (PMC6779298; doi:10.1371/journal.pone.0222799)
Supplement: S10 Table — (DOCX) [file pone.0222799.s010.docx]

**S10 Table. Synonymous Substitutions detected in the HCV core gene in Palestinian HCV isolates of subgenotype 4v (n=2).**

| **Substitution**  **nt** | **Substitution**  **aa** | **N** | **Reference** |
| --- | --- | --- | --- |
| T21C | P7P | 2 | KY627976 |
| C57A | P19P | 2 | N/A |
| G126A | P42P | 2 | N/A |
| A327G | P109P | 2 | JX227959 |
| C355T | L119L | 2 | JX227959 |

N: number of Palestinian isolates exhibiting the substitution.
